# Disrupted Gene Networks in Subfertile Hybrid House Mice

**DOI:** 10.1093/molbev/msaa002

**Published:** 2020-01-12

**Authors:** Katy Morgan, Bettina Harr, Michael A White, Bret A Payseur, Leslie M Turner

**Affiliations:** m1 Milner Centre for Evolution, Department of Biology and Biochemistry, University of Bath, Bath, United Kingdom; m2 Department of Evolutionary Genetics, Max Planck Institute for Evolutionary Biology, Plön, Germany; m3 Department of Genetics, University of Georgia, Athens, GE; m4 Laboratory of Genetics, University of Wisconsin, Madison, WI

**Keywords:** speciation, Dobzhansky–Muller incompatibilities, gene networks, *Mus musculus*, hybrid sterility, reproductive isolation

## Abstract

The Dobzhansky–Muller (DM) model provides a widely accepted mechanism for the evolution of reproductive isolation: incompatible substitutions disrupt interactions between genes. To date, few candidate incompatibility genes have been identified, leaving the genes driving speciation mostly uncharacterized. The importance of interactions in the DM model suggests that gene coexpression networks provide a powerful framework to understand disrupted pathways associated with postzygotic isolation. Here, we perform weighted gene coexpression network analysis to infer gene interactions in hybrids of two recently diverged European house mouse subspecies, *Mus mus domesticus* and *M. m. musculus*, which commonly show hybrid male sterility or subfertility. We use genome-wide testis expression data from 467 hybrid mice from two mapping populations: F_2_s from a laboratory cross between wild-derived pure subspecies strains and offspring of natural hybrids captured in the Central Europe hybrid zone. This large data set enabled us to build a robust consensus network using hybrid males with fertile phenotypes. We identify several expression modules, or groups of coexpressed genes, that are disrupted in subfertile hybrids, including modules functionally enriched for spermatogenesis, cilium and sperm flagellum organization, chromosome organization, and DNA repair, and including genes expressed in spermatogonia, spermatocytes, and spermatids. Our network-based approach enabled us to hone in on specific hub genes likely to be influencing module-wide gene expression and hence potentially driving large-effect DM incompatibilities. A disproportionate number of hub genes lie within sterility loci identified previously in the hybrid zone mapping population and represent promising candidate barrier genes and targets for future functional analysis.

## Introduction

According to the classic Dobzhansky–Muller (DM) model of speciation, mutations that accumulate independently and in different genomic regions may be incompatible when brought together in a hybrid background, resulting in disrupted epistasis and the development of postzygotic reproductive barriers (Muller 1942; Dobzhansky 1937). These barriers, which include reductions in hybrid fertility and/or hybrid viability, in turn restrict gene flow, enabling the further divergence of incipient species. The applicability of the DM model to allopatric speciation scenarios has been demonstrated through both theoretical and empirical studies (reviewed in Presgraves 2010). However, although many genes and loci influencing hybrid fertility have been described (reviewed in Maheshwari and Barbash 2011), characterizing the specific interactions between loci underpinning Dobzhansky-Muller incompatibilities (DMIs) remains a challenge.

Although DMIs were originally assumed to act independently of one another, such that individual loci are involved in a single DMI (Orr 1995), accumulating evidence raises questions about this assumption. A computational modeling study based on RNA folding demonstrated that as two populations evolve in allopatry, DMIs become increasingly complex over time (Kalirad and Azevedo 2017). In this context, the majority of DMIs could involve more than two loci and individual loci are expected to participate in multiple DMIs (Kalirad and Azevedo 2017). Evidence from empirical studies in house mice (Turner and Harr 2014; Turner et al. 2014), *Drosophila* (Phadnis 2011), as well as plant and fungal taxa, supports the prevalence of complex DMIs involving multiple partners (reviewed in Fraïsse et al. 2014). Theoretical studies have also implicated the role of divergence in complex regulatory pathways in driving postzygotic reproductive isolation via DMIs, with reproductive isolation being more likely to develop when regulatory pathways contain larger numbers of loci (Johnson and Porter 2000) and are under the influence of directional selection (Porter and Johnson 2002; Johnson and Porter 2006). Empirical evidence from house mice suggests that divergence in regulatory elements between subspecies disrupts epistatic interactions between sets of genes, resulting in significant reductions or elevations of gene expression in hybrid relative to pure subspecies individuals (Mack et al. 2016).

Misexpression in hybrid individuals is commonly observed and has been associated with reduced fertility in house mice (Good et al. 2010; Turner and Harr 2014; Turner et al. 2014; Mack et al. 2016; Larson et al. 2017), *Drosophila* (Michalak and Noor 2003; Moehring et al. 2007; Gomes and Civetta 2015), and cats (Davis et al. 2015). Understanding the role of potentially complex DMIs in driving patterns of misexpression may be facilitated by exploring gene interactions in a network context, in which expression patterns of sets of genes are allowed to depend upon one another. Network-based approaches cluster genes into coexpression modules, which are likely to be associated with common biological pathways and functions (e.g., Ayroles et al. 2009; Miller et al. 2010; reviewed in Mackay 2014), and have been employed to identify sets of genes associated with fitness-related phenotypes (Jumbo-Lucioni et al. 2010; Mack et al. 2018) and genes with disrupted interactions associated with disease (e.g., Saris et al. 2009; Miller et al. 2010; reviewed in De la Fuente 2010). Lee et al. (2013) demonstrated that SNPs associated with complex, polygenic traits were significantly more likely to lie within highly connected network “hub genes,” suggesting information regarding network topology may be useful in identifying biologically important genes. Network approaches have also been used to identify mechanisms involved in adaptive ecological divergence (Kelley et al. 2016; Gould et al. 2018; Zhao et al. 2019), and to explore the mechanisms underlying ecological speciation of sympatric lake whitefish ecotypes (Filteau et al. 2013). However, the power of network-based analyses for exploring the disrupted gene interactions associated with reduced hybrid fertility and postzygotic reproductive isolation is yet to be realized.

As mutations and incompatibilities continue to accumulate between independently evolving lineages over time, identifying the incompatibilities that initially caused reproductive isolation requires studying recently diverged lineages, with incomplete reproductive barriers. The European house mouse (*Mus musculus*) provides one such system. Three subspecies, *M. m. musculus*, *M. m. domesticus*, and *M. m. castaneus*, diverged ∼500,000 years ago (Geraldes et al. 2011). Following this initial divergence, *M. m. musculus* and *M. m. domesticus* (henceforth referred to as *musculus* and *domesticus*, respectively) used different routes to colonize Europe providing the opportunity for the accumulation of DMIs in allopatry. Laboratory crosses between *musculus* and *domesticus* have demonstrated reduced fertility in hybrid males (Forejt and Ivanyi 1974; Oka et al. 2004; Good, Dean, et al. 2008), although the degree of sterility varies depending on the nature of the cross (Britton-Davidian et al. 2005; Vyskočilová et al. 2005; Good, Handel, et al. 2008; Larson et al. 2018). Genetic studies of *musculus–domesticus* hybrids have also led to the first characterization of a mammalian hybrid incompatibility gene, *Prdm9* (Mihola et al. 2009), an autosomal histone methyltransferase which binds DNA at recombination hotspots and plays an important role in the initiation of meiotic recombination (Baudat et al. 2010; Davies et al. 2016). Negative interactions between some *domesticus Prdm9* alleles and loci on the *musculus* X-chromosome disrupt expression of the X-chromosome and chromosome synapsis resulting in meiotic arrest (Bhattacharyya et al. 2013, 2014; Campbell et al. 2013; Larson et al. 2017).

Following colonization of Europe, *musculus* and *domesticus* expanded their ranges to form a zone of secondary contact traversing Central Europe (Macholán et al. 2003; Janoušek et al. 2012). Hybridization along this secondary contact zone is common resulting in a cline of genomic admixture (Payseur et al. 2004; Macholán et al. 2007; Teeter et al. 2007; Janoušek et al. 2012). Reduced male fertility is frequent in wild-caught hybrids, but complete sterility is rare (Britton-Davidian et al. 2005; Albrechtova et al. 2012; Turner et al. 2012), suggesting that this postzygotic barrier reduces gene flow and contributes to maintenance of the subspecies boundary, but is incomplete and variable in strength across populations.

In the present study, we are building on genetic mapping of fertility phenotypes and gene expression traits in F_2_*musculus–domesticus* hybrids generated through a laboratory cross (White et al. 2011; Turner et al. 2014), and in offspring of wild-caught *musculus–domesticus* hybrids (Turner and Harr 2014), which have identified many autosomal and X-linked sterility loci. DMIs were mapped in both studies, using different methods, yet many loci and interactions are shared between mapping populations (Turner and Harr 2014). Most loci have multiple interaction partners supporting the presence of complex DMIs (Turner and Harr 2014; Turner et al. 2014). Identifying the underlying mechanisms and causative genes remains a challenge, because many loci encompass a large number of genes.

Here, we characterize disruptions in gene networks associated with hybrid male sterility in mice by analyzing patterns of gene coexpression using microarray data from a total of 467 mice from two *musculus–domesticus* hybrid mapping populations (Turner and Harr 2014; Turner et al. 2014). We use weighted gene coexpression analysis (WGCNA; Langfelder and Horvath 2008) to: 1) characterize a consensus “fertile” network, 2) identify modules of coexpressed genes associated with biological pathways and processes, 3) identify modules disrupted in subfertile hybrids, and 4) identify specific candidate genes likely to be driving network disruptions in subfertile hybrids.

## Results

### Concordant Genome-Wide Testis Expression Patterns in F_2_ and Hybrid Zone Hybrids

We analyzed genome-wide expression patterns in testes from 295 F_2_ hybrids between two inbred strains of *musculus* and *domesticus* (White et al. 2011; Turner et al. 2014) and 172 lab-bred male offspring of mice wild-caught from a hybrid zone (HZ) (Turner and Harr 2014). Principal components analysis (PCA) of the F_2_ and HZ data sets showed similar overall patterns (supplementary fig. 1, Supplementary Material online). For both populations, PC1, which explains 20.1% of variation in F_2_ hybrids and 27.8% of variation in HZ hybrids, is clearly associated with fertility classified on the basis of relative testis weight (testis weight/body weight) and sperm count (White et al. 2011; Turner et al. 2012; supplementary fig. 1*A* and *B*, Supplementary Material online). The PC1 loadings for probes in the two data sets are strikingly highly correlated (*r* = 0.91, *P* < 2.2e-16) suggesting a common set of genes contribute to the “sterile” expression pattern (supplementary fig. 1*C*, Supplementary Material online). The remarkable similarities in overall testis expression pattern between mapping populations, and previous evidence for shared incompatibilities (Turner and Harr 2014), motivated us to combine expression data from F_2_ and HZ hybrids to characterize gene network disruptions associated with sterility.

**Fig. 1.**
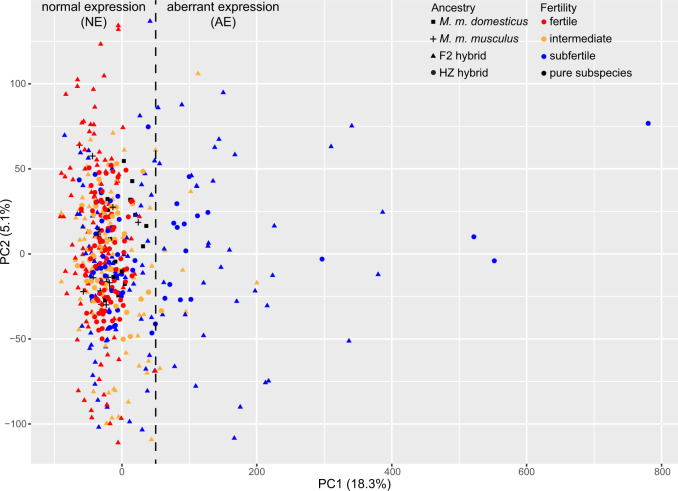
Principal components analysis (PCA) of genome-wide expression in testis of pure *Mus musculus domesticus*, *M. m. musculus* and hybrids, PC1 versus PC2. Point shape indicates subspecies or hybrid mapping population for each individual and point color indicates fertility class (see Materials and Methods). Subfertile hybrids with PC1 scores outside the range observed in pure subspecies males and fertile hybrids are classified as “Subfertile Aberrant Expression” (SFAE), whereas subfertile hybrids within the pure subspecies and fertile hybrid range were classified as “Subfertile Normal Expression” (SFNE). The dashed line indicates the cutoff between SFNE and SFAE hybrid groups.

After combining and batch-correcting data for the 36,896 probes (representing 17,775 unique Entrez genes) with above-background levels of expression from F_2_ and HZ hybrid and pure subspecies males, PC1 explains 18.26% of variation in gene expression (fig. 1) and is significantly negatively correlated with both relative testis weight and sperm count (*r* = −0.527 and −0.161, *P* = 2.2e-16 and 3.3e-4, respectively). The PC1 scores of fertile hybrid and pure subspecies males show similar levels of variation (variance: 609.36 and 616.10, respectively), whereas variation in PC1 scores is considerably higher in hybrids with subfertile phenotypes (variance: 15,149.70). Hybrids for which both fertility phenotypes fall within the pure subspecies range, yet at least one of the phenotypes is >1 SD from the pure subspecies mean, were classified as “intermediate phenotype” (see Materials and Methods) and show intermediate levels of variation along PC1 (variance: 1,816.78).

A sizeable proportion (33.5%) of the subfertile hybrids have PC1 scores that exceed the fertile hybrid and pure subspecies range (fig. 1); we classified these individuals as “Subfertile Aberrant Expression” (SFAE), and classified subfertile hybrids that cluster with fertile hybrids and pure subspecies males along PC1 as “Subfertile Normal Expression” (SFNE). A total of 69 F_2_ and 38 HZ hybrids were categorized as SFNE, whereas 37 F_2_ and 17 HZ hybrids were categorized as SFAE (fig. 1). PC3, which explains 4.28% of variation, is associated with subspecies ancestry: *musculus* individuals have high scores, *domesticus* individuals have low scores, and hybrids have intermediate scores (supplementary fig. 2, Supplementary Material online).

### Fertile Hybrid Consensus Network

**Fig. 2.**
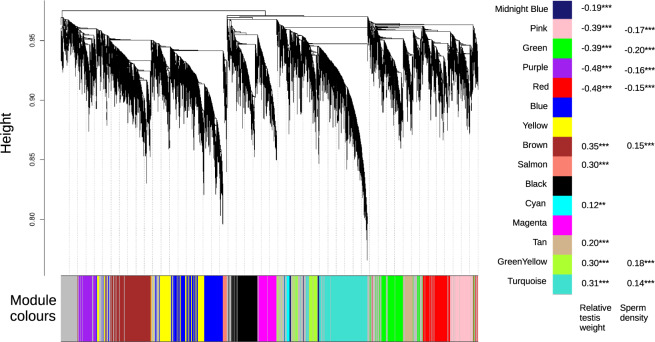
Gene coexpression modules. (*A*) Consensus fertile network generated using weighted gene coexpression network analysis (WGCNA; Langfelder and Horvath 2008) of testis expression from 102 fertile F_2_ and 79 fertile HZ hybrid males. The dendrogram shows the clustering of probes based on the topological overlap distance within fertile hybrids. Color bar beneath the dendrogram indicates coexpression modules. (*B*) The correlation between the module eigengene (ME), representing overall module expression, and sterility phenotypes. Significant positive correlations are indicated in red and significant negative correlations are indicated in blue; **P < 0.01, ***P < 0.001.

To identify potentially interacting sets of genes that are coexpressed in fertile hybrids from different genomic backgrounds, we constructed a consensus fertile network using expression data from the fertile F_2_ (*n* = 102) and HZ (*n* = 79) hybrids and 18,411 probes representing 10,531 genes (see Materials and Methods for details). A total of 14,346 probes, representing 7,989 unique genes, were assigned to 1 of the 15 coexpression modules (fig. 2*A* andsupplementary data 1, Supplementary Material online); 4,065 probes could not be assigned to a module and are shown in the gray, “bin” module. Thirteen modules are significantly enriched for specific functions on the basis of Gene Ontology (GO) analysis, of which seven were significantly enriched for spermatogenesis or potentially related functions (table 1 and supplementary data 2, Supplementary Material online). The module eigengene (ME), which describes the overall expression level of each module across the full data set of fertile and subfertile hybrids, was significantly positively correlated with both sperm count and relative testis weight for three modules, and significantly positively correlated with relative testis weight alone for an additional three modules (fig. 2*B*). Four modules have an ME which is significantly negatively correlated with both sterility phenotypes, whereas the ME of one additional module is significantly negatively correlated with relative testis weight alone. Of the seven modules that are significantly enriched for spermatogenesis or potentially related functions, two have an ME which is significantly positively correlated with at least one of the fertility phenotypes (Brown and Tan), whereas two have an ME which is significantly negatively correlated with at least one of the fertility phenotypes (Green and Pink) (fig. 2*B*). These correlations suggest that the inferred modules of coexpression are informative about fertility.

**Table 1.** Module Enrichment within the Fertile F_2_ and HZ Networks, Using the Testis-Expressed Genes As a Background.

Network Module	Number of Genes	Most Significant GO Enrichment Term			Testis Cell Type(s) in Which a Significant Proportion of Genes are Expressed^b^
		GO Term	Number Genes in GO Term	*P* Value^a^	
Black	719	Ubiquitin-like protein transferase activity	44	3.4e-7	SG, eP1, Sertoli
Blue	1,421	GTPase binding	88	1.2e-11	SG, eP1, mP, MII, S1, S11, Sertoli
		**Spermatogenesis**	72	1.1e-4	
Brown	1,274	Ubiquitin-dependent protein catabolic process	74	1.4e-4	SG, D, MI, MII, S11, Sertoli
		**Ciliary part**	50	4.6e-2	
Cyan	102	—	—	—	
Green	894	Protein ubiquitination	57	2.2e-4	SG, eP1, S1, Sertoli
		**Chromosome segregation**	31	1.7e-2	
Greenyellow	455	—	—	—	S8
Magenta	602	Nuclear chromosome	55	1.3e-10	SG, eP1, eP2, MII, Sertoli
		**Regulation of chromosome organization**	38	1.8e-7	
Midnightblue	90	Phagocytosis, engulfment	5	6.3e-3	
Pink	571	Mitochondrial protein complex	50	1.7e-22	SG, Leydig
		**Mitochondrial respiratory chain**	17	4.3e-11	
Purple	435	Monocarboxylic acid metabolic process	38	9.3e-6	Leydig
Red	717	Monocarboxylic acid metabolic process	54	5.8e-7	Leydig, Sertoli
Salmon	203	Microtubule	16	4.5e-3	SG, S11
Tan	320	Ribosome	11	1.8e-2	SG
		**Spermatogenesis**	22	4.3e-2	
Turquoise	1,436	Olfactory receptor activity	139	9.9e-39	
Yellow	933	mRNA processing	50	6.4e-5	SG, eP1, eP2, MII, S1, Sertoli
		**Spermatogenesis**	49	4.6e-3	

Note.—GOs potentially related to spermatogenesis are listed in bold.

a *P* values were corrected using the Benjamini–Hochberg correction (Benjamini and Hochberg 1995).

b Significant overlaps between genes expressed in specific testis cell types (Ernst et al. 2019) and module gene content were identified using Fisher’s exact tests with Benjamini–Hochberg correction for multiple tests, using all testis-expressed genes as the background. Testis cell types include spermatogonia (SG), early pachytene spermatocytes (eP1 and eP2, respectively), diplotene spermatocytes (D), metaphase I and II spermatocytes (MI and MII, respectively), stage 1–11 spermatids (S1–11), and Sertoli and Leydig cells.

In addition to the two fertility traits used to classify hybrid males, data describing several sperm motility traits were also available for a subset of the HZ males (*n* = 121) (Turner et al. 2012). Interestingly, the ME of the Brown, Greenyellow, and Pink modules were all significantly positively correlated with beat-cross frequency (BCF) (*r*=0.19, 0.36, and 0.23, *P* = 0.034, 5.9e-5, and 0.012, respectively), which is defined as the frequency with which the sperm head crosses the middle plane of the straight trajectory (Turner et al. 2012). In human sperm, BCF is roughly proportional to the frequency with which the sperm flagellum beats (Serres et al. 1984). This is of note since the Brown module is enriched for genes expressed in the ciliary part (*P* = 0.049), likely to be important for the functioning of the sperm flagellum, and the Pink module is enriched for genes involved in the mitochondrial respiratory chain (*P* = 4.3e-11), which is highly active in motile sperm (Amaral et al. 2013).

### Network Preservation in Subfertile Hybrids

**Fig. 3.**
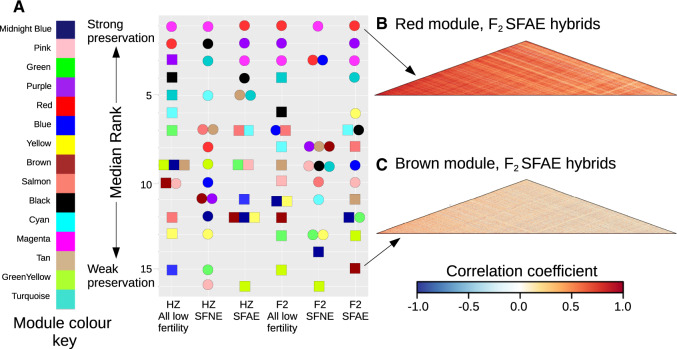
Preservation of the fertile consensus network within subfertile hybrids classified according to mapping population derived and expression profile (SFNE or SFAE, see fig. 1). (*A*) Module preservation estimated using median rank statistics. Color indicates module identity. Circles represent significantly preserved modules, and squares represent modules not significantly preserved. (*B* and *C*) Coexpression heatmaps for examples of well-preserved (Red) and poorly preserved (Brown) modules. Heatmaps show pairwise correlations between expression values for all genes within modules for SFAE F_2_ hybrids.

To determine whether coexpression networks are disrupted in subfertile and intermediate phenotype hybrids, we estimated module preservation using two approaches based on a set of metrics developed by Langfelder et al. (2011; see Materials and Methods). Module preservation was assessed independently for F_2_ and HZ hybrids, because the presence and prevalence of specific DMIs and associated network disruptions may vary between mapping populations. Although significant preservation was detected for all modules in intermediate phenotype hybrid groups, several modules showed a lack of significant preservation in the subfertile phenotype hybrids (see supplementary table 1, Supplementary Material online). Figure 3*A* shows the estimated preservation of each module from the consensus fertile network in F_2_ and HZ hybrids with subfertile phenotypes and either aberrant or normal overall expression patterns. Note that median rank shows relative preservation within a group, whereas *Z* statistics allow for comparison between groups. Modules showing significant evidence for preservation in subfertile hybrids, according to the preservation statistics presented in supplementary table 1, Supplementary Material online, are represented by circles, whereas modules showing a lack of significant preservation are represented by squares (fig. 3*A*). Figure 3*B* and *C* illustrate coexpression patterns within a well preserved (Red) versus a poorly preserved (Brown) module in the F_2_ SFAE hybrids. Pairwise coexpression in the Red module is characterized by strong positive correlations (fig. 3*B*). In contrast, many pairwise expression correlations in the Brown module are weakened or even reversed in direction (fig. 3*C*), suggesting substantial disruption in the coexpression of these gene pairs.

The level of preservation of modules from the fertile network was similar in subfertile HZ and F_2_ hybrids. Three modules (Magenta, Red, Pink) showed strong evidence for preservation in subfertile HZ hybrids (fig. 3*A* andsupplementary table 1, Supplementary Material online); the remaining 12 modules had either a *Z*_summary_ <10 and/or nonsignificant NetRep statistics, indicating weak or a lack of preservation (Langfelder et al. 2011; Ritchie et al. 2016, see Materials and Methods). In F_2_ hybrids, four modules (Magenta, Red, Purple, and Blue) showed strong evidence for preservation in subfertile mice.

As expected, module preservation was much higher in subfertile hybrids with normal expression based on PC1 (SFNE); all modules showed strong evidence of preservation in the HZ SFNE hybrids, and 13 out of 15 modules were strongly preserved in F_2_ SFNE hybrids (Greenyellow and Midnightblue modules had nonsignificant NetRep scores; supplementary table 1, Supplementary Material online). By contrast, preservation was lower in subfertile hybrids with aberrant expression; 6 out of 15 modules were preserved in HZ SFAE hybrids, and 9 modules in F_2_ SFAE hybrids.

Many modules show consistent levels of preservation across subfertile hybrid classes (fig. 3*A*). The Red, Purple, Magenta, and Turquoise modules consistently rank among the most strongly preserved modules; these modules are significantly enriched for metabolic processes, histone binding, and olfactory receptor activity, respectively (table 1). By contrast, the Brown, Green, Yellow, Midnightblue, and Greenyellow modules consistently rank among the modules with the weakest preservation; these modules are significantly enriched for histone binding and the ciliary part, DNA repair and chromosome segregation, mRNA processing and spermatogenesis, and phagocytosis (the Greenyellow module has no significant enrichments). Notably, although the Magenta and Green modules are significantly enriched for similar processes (chromosome organization and segregation, respectively), the Magenta module appears to be one of the best-preserved modules, whereas the Green module is among the least preserved across hybrid groups (fig. 3*A*).

Although consistencies across subfertile groups are apparent, notable differences were also detected between the HZ and F_2_ hybrids. For example, the Blue module, enriched for spermatogenesis, is significantly preserved in the F_2_ SFAE hybrids, yet shows relatively poor preservation in the HZ SFAE hybrids (fig. 3*A* and see supplementary table 1, Supplementary Material online). The Yellow module, which is also significantly enriched for spermatogenesis, is among the least preserved modules in all subfertile hybrid groups except for the F_2_ SFAE, within which it is relatively well preserved.

In summary, broad similarity of module preservation statistics across subfertile classes provides further evidence that specific functions and pathways are commonly disrupted in sterile hybrids. By contrast, modules showing differences in conservation in subfertile HZ versus F_2_ hybrids suggest some network disruptions are unique to specific mapping populations.

### Differentially Correlated Genes

Our next aim was to identify specific genes responsible for the signal interpreted as network disruption in subfertile hybrids. We identified genes with significant changes in coexpression pattern in subfertile versus fertile hybrids using differential correlation analysis, which was performed independently for each module using the DGCA R package (McKenzie et al. 2016). A total of 2,800 genes showed a significant loss or reversal of coexpression pattern within at least one of the subfertile groups (supplementary data 1, Supplementary Material online). Although the power to detect changes in coexpression patterns is likely to differ between subfertile groups due to varying sample size, differentially correlated genes were detected within all four subfertile hybrid groups (SFNE and SFAE F_2_ and HZ hybrids). As expected, a higher proportion of genes were differentially correlated in weakly preserved (28.89–46.59%) versus strongly preserved (1.12–3.65%) modules (supplementary table 2, Supplementary Material online).

### Modules Associated with Specific Stages of Spermatogenesis or Testis Cell Types

To determine whether coexpression modules are associated with specific stages of spermatogenesis, we tested for significant enrichment of genes expressed in different testis cell types, which was recently determined at high resolution using a combination of single-cell RNAseq and bulk RNAseq at different time points during the first stage of spermatogenesis (gene lists from Supplementary Data 2 in Ernst et al. 2019). The gene content of nine modules (Black, Blue, Brown, Green, Magenta, Pink, Salmon, Tan, and Yellow) overlaps significantly with genes expressed in spermatogonia (table 1). Six modules (Black, Blue, Brown, Green, Magenta, and Yellow) overlap with genes expressed in spermatocytes during stages of meiosis (early pachytene, diplotene, and metaphase). Six modules (Blue, Brown, Green, Greenyellow, Salmon, and Yellow) overlap with genes expressed postmeiotically in at least 1 of the 11 stages of developing spermatids. For somatic cell types, seven modules (Black, Blue, Brown, Green, Magenta, Red, Yellow) overlap with genes expressed in Sertoli cells and three modules (Pink, Purple, and Red) are enriched for genes expressed in Leydig cells. Thus, of the 15 modules, 3 overlap significantly with genes expressed during spermatogenesis only (Greenyellow, Salmon, and Tan), 7 modules overlap significantly with both genes expressed in spermatogenesis and with genes expressed in somatic cells (Black, Blue, Brown, Green, Magenta, Pink, and Yellow), 2 modules overlap only with somatic cells (Purple and Red), and 3 modules do not overlap with genes expressed in any of specific testis cell types (Cyan, Midnightblue, and Turquoise). The complete lists of genes with cell type-specific expression (Ernst et al. 2019) are in supplementary data 3, Supplementary Material online. Our findings indicate that several coexpression modules can be linked with one or more stages of the spermatogenesis process.

### Modules Associated with *trans*-eQTL Hotspots

Turner et al. (2014) identified 11 *trans*-expression quantitative trait loci (eQTL) hotspots, that is, regions of the genome with significant effects on the expression of hundreds to thousands of quantitative trait transcripts (QTTs) in F_2_ hybrids, and provided several lines of evidence linking these hotspots to sterility phenotypes. We investigated whether *trans*-eQTL hotspots affected specific parts of the gene coexpression network by testing for overlap between QTT associated with each hotspot and genes in modules from the fertile network. Genes in seven modules overlap with QTT from one to seven hotspots (table 2). Modules negatively correlated with relative testis weight and/or sperm count (Green, Red, Purple) overlap significantly with QTT showing high expression associated with the sterile allele, as established from expression patterns in sterile F_1_ hybrids (Turner et al. 2014), whereas modules positively correlated with fertility phenotypes (Brown, Greenyellow, Turquoise) overlap QTT with low expression associated with the sterile allele. That is, in all the cases, “sterile” versus “fertile” patterns were consistent between coexpression modules and QTT associated with sterile versus fertile alleles.

**Table 2.** Significant Overlap between Genes within Coexpression Modules and Quantitative Trait Transcripts (QTT) Associated with *trans*-eQTL Hotspots (Turner et al. 2014).

Coexpression Module	*trans*-eQTL Hotspot, Direction^a^	Sterile Expression Pattern^b^	Number of Genes in Module	Number of QTT	Overlap between Gene Sets	Fisher’s Exact *P* Value	Corrected *P* Value^c^
Black	18–38 cM Chr 15, Mhigh	Low	719	511	62	1.1e-5	5.1e-5
Brown	38–44 cM Chr 3, Dhigh	Low	1,271	102	30	3.2e-6	4.8e-5
Green	18–38 cM Chr 15, Dhigh	High	894	867	102	7.2e-4	3.6e-3
Greenyellow	18–38 cM Chr 15, Mhigh	Low	455	511	47	1.2e-6	8.4e-6
Red	26–38 cM Chr 2, Dhigh	High	717	347	85	7.1e-26	1.1e-24
	54–62 cM Chr 11, Dhigh	High	717	763	166	5.1e-44	7.1e-43
	18–38 cM Chr 15, Dhigh	High	717	246	59	1.1e-17	8.5e-17
	46–50 cM Chr 15, Dhigh	High	717	867	293	3.5e-140	5.2e-139
	0–16 cM Chr 17, Mhigh	High	717	100	31	4.7e-13	4.7e-13
	0–42 cM X Chr, Mhigh	High	717	1,117	171	3.3e-25	2.5e-24
Purple	26–38 cM Chr 2, Dhigh	High	435	347	36	5.1e-7	3.8e-6
	4–24 cM Chr 10, Dhigh	High	435	763	88	7.7e-19	5.4e-18
	54–62 cM Chr 11, Dhigh	High	435	246	58	5.2e-28	8.8e-27
	18–38 cM Chr 15, Dhigh	High	435	867	130	1.0e-40	7.5e-40
	46–50 cM Chr 15, Dhigh	High	435	100	14	7.4e-5	5.6e-4
	0–16 cM Chr 17, Mhigh	High	435	1,117	155	3.3e-45	5.0e-44
	0–42 cM X Chr, Mhigh	High	435	3,329	238	6.2e-23	4.7e-22
Turquoise	18–38 cM Chr 15, Mhigh	Low	1,434	511	115	4.8e-8	6.7e-7

a Chromosome and cM position for *trans*-eQTL hotspots, as reported in Turner et al. (2014), and subset of QTTs associated with either the *domesticus* (Dhigh) or *musculus* (Mhigh) eQTL allele.

b QTTs showed higher or lower expression associated with the “sterile” eQTL allele (classified on the basis of multiple criteria, see Table 2 in Turner et al. 2014).

c Benjamini–Hochberg correction for multiple tests.

Modules with higher preservation in subfertile hybrids (Red, Purple) overlap with QTT associated with multiple *trans*-eQTL hotspots (table 2), highlighting potential interactions between eQTL hotspots. In contrast, modules with intermediate (Black, Turquoise) or weak (Brown, Green, and Greenyellow) preservation in subfertile hybrids each overlap significantly with QTT associated with a single *trans*-eQTL hotspot. In summary, five modules with intermediate or weak preservation overlap significantly with QTT associated with a specific *trans*-eQTL hotspot supporting an influence of the underlying sterility alleles on specific parts of the fertile gene network.

### Module Hub Genes

We next identified hub genes in the fertile network. Hub genes are highly connected within modules, and thus are likely to have a disproportionate effect on module-wide expression patterns. We identified 281 hub genes across the 15 modules on the basis of degree (number of connections) and module membership (correlation between the expression of a gene and the ME) (Horvath and Dong 2008). Of the 281 hub genes, 95 genes within 10 modules show a significant loss or reversal of coexpression pattern in at least one of the subfertile hybrid groups relative to the fertile hybrids (table 3). Of those 95 differentially correlated hub genes, 29 and 57 show a significant loss of coexpression pattern in the SFAE and SFNE F_2_, respectively, whereas 11 and 10 show a significant loss of coexpression pattern the SFAE and SFNE HZ, respectively (supplementary data 4, Supplementary Material online). Hence, although the WGCNA and NetRep analyses show evidence for stronger module preservation in SFNE relative to SFAE hybrids, differential correlation analysis shows evidence for the disruption of module hub gene interactions in both the SFAE and SFNE hybrid groups.

**Table 3.** Candidate Module Hub Genes.

Module	Hub Genes
Black	*1700081L11Rik*, *Asxl2*^a^, *Atxn2*^b^, *Fto*^b^^,^^c^, *Gle1*^b^^,^^c^, *Hectd2*, *Lpp*^b^, *Nr2c2*^a^^,^^b^^,^^d^, *Rab14*^b^^,^^c^, *Sbno1*^a^^,^^b^, *Sltm*^a^^,^^b^, *Srpk2*^a^^,^^b^, *Stim1*, *Pibf1*^b^, *Ppp6r3*, *Ube2e2*, *Znrf3*^b^
Blue	***1700029G01Rik*** ^b^, ***Agpat6***^b^, ***Akap1***^b^^,^^d^, ***Ankrd13a***^b^, ***Ccdc147***^b^, ***Dnhd1***^b^, ***Gm101***^b^, ***Gm14857***^b^, ***Hira***^a^^,^^b^, ***Hk1***^b^^,^^d^, ***Inpp5e***^b^^,^^c^, ***Nupl2***^a^^,^^b^, ***Phf12***^a^^,^^b^, ***Rangap1***^b^^,^^d^, ***Rnf19b***^b^, ***Sbf2***^b^, ***Slain2***^b^, ***Sort***^a^^,^^b^, ***Triml1***^b^, ***Zfp445***^a^, *1110037F02Rik*, *1700029F09Rik*^b^, *4930473A06Rik*^b^, *4932425I24Rik*^b^, *Adam1a*^a^^,^^b^, *Ankrd40*^d^, *Brap*^b^, *Cnot1*^a^^,^^b^^,^^c^, *Cttn*, *Cpsf2*^b^, *D1Pas1*^a^, *Fam48a*^b^, *Gigyf1*^b^, *Ggcx*, *Hsp90ab1*^b^^,^^c^, *Ift172*^b^, *Insig2*^b^, *Ints10*^b^, *Ipo11*, *Kif5b*, *Mtmr9*^b^, *Mum1*^b^, *Nasp*^b^, *N4bp1*^b^, *Nelf*^b^^,^^c^, *Net1*^b^, *Nxf1*^a^, *Pfas*^b^^,^^c^, *Ranbp10*, *Rnd3*, *Sarm1*^b^, *Shprh*, *Smg5*^b^^,^^d^, *Snx27*^d^, *Spag5*, *Tapt1*, *Tex2*^b^, *Trpc4ap*^b^, *Usp33*, *Zfp541*^a^
Brown	***Dbnl*** ^b^, ***Evi5l***^b^, ***Fkbp4***^b^, ***Kif17***^b^, ***Pacs2***^b^, ***Prkcd***^b^^,^^c^, ***Ptdss2***^b^, ***Smarcd1***, ***Trafd1***^b^, ***Xpo6***
Cyan	***Foxl2*** ^a^, *Arhgap20*, *N4bp2l2*^b^, *Olfr1350*, *Plekha5*^b^, *Wac*^b^
Green	***Clint1*** ^b^, ***Dpy19l4***, ***Maml1***^a^, ***Pik3c2a***, ***Zfp711***^a^^,^^b^^,^^d^, ***Zfp770***^a^^,^^b^^,^^d^, *Dld*^a^^,^^b^^,^^c^, *Ganc*^b^
Greenyellow	***D10Bwg1379e***, ***E130304I02Rik***, ***Fbxo2***^b^, ***Mapkap1***^b^^,^^c^, ***Olfr279***, ***Rapgef1***^b^^,^^c^, ***Shc2***, ***Sprr2b***^d^, ***Tbc1d2***^b^
Magenta	***4921528I01Rik***, *B230208H17Rik*^c^, *Rab6l*^c^, *Akap13*^b^, *Casc5*^b^^,^^d^, *Cdk5rap2*^a^^,^^b^, *Cep290*^a^^,^^b^, *Chd4*^a^^,^^b^, *Ckap5*^b^^,^^d^, *Clasp1*, *Cul5*^b^, *Ddx21*^a^^,^^b^^,^^d^, *Ddx46*^b^, *Dek*^b^, *Dicer1*^a^^,^^b^, *Eea1*^b^, *Eif5b*^b^, *Eif3c*^b^, *Eprs*^b^, *Golgb1*, *Gpatch8*, *Hcfc1*^d^, *Heatr6*^b^^,^^d^, *Hectd1*^b^^,^^c^, *Kdm5b*^a^^,^^b^, *Kif20b*^b^, *Man1a2*^d^, *Mlh3*^a^^,^^b^, *Nol8*^b^, *Nvl*^b^^,^^c^, *Parp10*^b^^,^^d^, *Pcm1*^b^, *Ppp2r5e*^b^, *Prpf40a*^b^, *R3hdm1*, *Rapgef6*^b^, *Rif1*^a^^,^^b^, *Rock1*^b^, *Rrbp1*, *Setd5*, *Sfrs18*, *Smc2*^a^^,^^b^, *Spnb2*, *Stlm*^a^^,^^b^, *Thoc2*^a^^,^^d^, *Thrap3*^a^^,^^b^, *Tnrc18*^b^, *Tpr*^a^^,^^b^, *Trip11*^b^, *Ubxn4*^b^, *Wasf2*^b^, *Wapal*^a^^,^^b^, *Zc3h13*^b^
Midnightblue	***Adam28*** ^c^, ***Hoxb6***^a^^,^^d^, ***Krt10***^b^, ***Lcn9***^c^, *Cst11*, *Cyp4a12a*, *Lcn10*^c^, *Ly6g5c*^d^, *Krt10*, *Krt14*, *Rnf186*, *Serpinf2*
Pink	***1810027O10Rik***, *Bola2*^b^, *Cox17*, *Med31*^a^^,^^b^, *Pop5*^b^, *Pop7*^b^, *Ucqr*
Purple	*Cry2* ^a^ ^,^ ^d^, *Frk*^a^, *Gart*, *Glb1l2*, *Gnas*^a^, *Mboat1*, *Kcnk1*, *Zfp185*^b^^,^^c^^,^^d^
Red	*Acaa2*, *Chst8*^c^, *Ifnar2*^a^, *Itm2b*^c^, *Gpx1*^a^, *Cyp11a1*^c^, *Rnf128*^b^^,^^c^^,^^d^, *Tmem30b*
Salmon	*Arhgef2* ^b^ ^,^ ^d^, *Fam188b*^b^, *Fgl1*^b^, *Gm7416*^d^, *Itgb3*^b^, *Krtap3-1*, *Pomt2*^b^, *Slc11a2*
Tan	*Cops2* ^a^ ^,^ ^b^, *Oxr1*^b^, *Ralgapa1*^a^^,^^b^, *Tmem30a*
Turquoise	***2410017I17Rik*** ^d^, ***A730081D07Rik***, ***Adamts14***^d^, ***Atp8b5***^b^, ***Atpif1***, ***Calcoco1***^a^, ***Chst11***, ***Csf2rb***^d^, ***Fam155a***, ***Gm10229***, ***Gm13033***, ***Gpr152***, ***Gsx1***^a^^,^^d^, ***Helq***^b^, ***Ifna13***, ***Lce1l***^d^, ***Lrrc8e***^b^, ***Mc3r***, ***Mis18bp1***^b^, ***Mlxip***^a^^,^^b^, ***Msx1***^a^, ***Ndor1***^b^^,^^d^, ***Olfr1131***^d^, ***Olfr166***, ***Olfr414***^c^, ***Olfr656***, ***Osr1***^a^^,^^c^, ***Phxr4***^b^, ***Prune2***, ***Rasgrp4***^b^, ***Rsl1d1***^a^^,^^b^^,^^c^, ***Slc22a8***, ***Slco2a1***^b^, ***Speg***, ***Sprr2e***^d^, ***Sprr2j-ps***^d^, ***Tmc8***, ***Vmn1r192***, *Ctrl*, *Cyp2d34*^d^, *Gja5*^d^, *Gm10319*, *Gm11019*, *Helq*^b^, *Mdga2*^b^, *Mfap2*^b^, *Mlxip*^b^, *Ndor*^b^^,^^c^, *Nlrp3*, *Olfr374*, *Olfr434*, *Olfr549*, *Olfr577*, *Olfr60*, *Sall4*^a^^,^^b^, *Serhl*^b^^,^^d^, *Tbkbp1*^b^^,^^d^, *Tmem8c*^c^
Yellow	***Arl8b*** ^b^, ***Atr***^b^, ***Gm9805***^d^, ***Gmps***^b^, ***Lnp***^b^, ***Tcerg1***^a^, *Aars*^a^^,^^b^^,^^c^, *Nom1*^b^, *Pex13*^b^, *Psmd2*^b^, *Rab5a*^b^^,^^c^, *Scaf11*^d^

Note.—Differentially correlated hub genes, for which patterns of coexpression detected in fertile hybrids are significantly lost or reversed in at least one subfertile hybrid group, are highlighted in bold.

a Genes with GO terms related to regulation of gene expression and/or male fertility.

b Genes that have been found to be expressed in one or more class of testis germ cell (Ernst et al. 2019).

c Genes within sterility loci identified by GWAS in HZ hybrids (Turner and Harr 2014).

d Genes found within *trans*-eQTL hotspots (Turner et al. 2014).

Table 3 lists the module hub genes indicating the following as potential candidates for DMIs: genes with different coexpression patterns in subfertile relative to fertile hybrids (*n* = 95); genes that have been found to be expressed within spermatogonia, spermatocytes, or spermatids (Ernst et al. 2019) (*n* = 152); genes with GOs including those related to the regulation of gene expression and/or male fertility (*n* = 52); genes that fall within previously identified *trans*-eQTL hotspots (Turner et al. 2014) (*n* = 40); and genes within sterility loci identified by GWAS in HZ mice (Turner and Harr 2014) (*n* = 31). Permutation tests revealed that the module hubs include a significantly larger number of genes that fall within GWAS sterility regions than do random draws from all testis-expressed genes (*P* = 0.04) and from all module genes (genes assigned to 1 of the 15 modules in the consensus fertile network, *P* = 0.05), supporting an enrichment for genes within GWAS sterility regions in the module hubs. Although the number of genes within eQTL hotspots did not differ significantly between the module hub genes and random draws from the testis-expressed genes (*P* = 0.50) and from all module genes (*P* = 0.65), this result is not unexpected as the eQTL hotspot regions span large regions of the genome, and likely contain many genes that play no role in fertility.

**Fig. 4.**
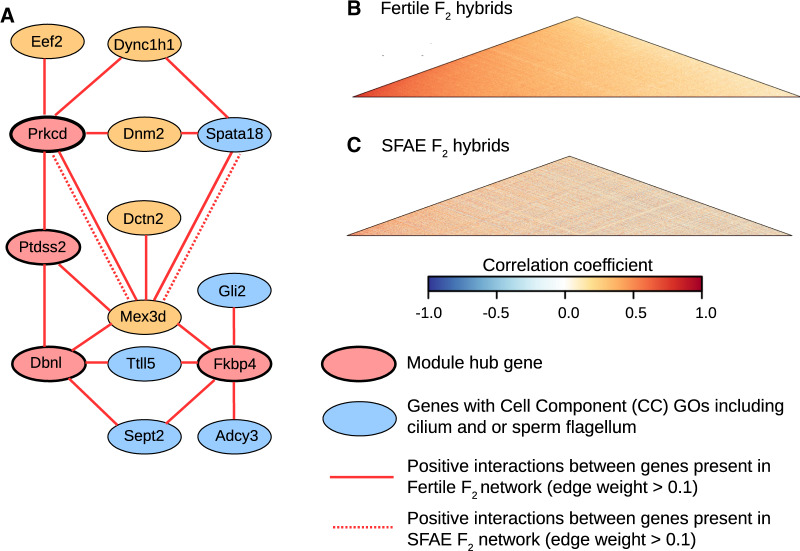
Disrupted interactions of Brown module genes in subfertile F_2_ hybrids. (*A*) Interactions between Brown module hub genes (red nodes) and genes with GOs including cilium/and or sperm flagellum (blue nodes) in fertile and SFAE F_2_ hybrids. Orange nodes indicate intermediate genes with functions potentially related to male fertility. Gene interactions with an edge-weight exceeding 0.1, as estimated using topological overlap matrices, are indicated using continuous and dashed lines for the fertile and SFAE hybrids, respectively. (*B* and *C*) Coexpression heatmaps showing pairwise correlations between expression values of Brown module genes in fertile and SFAE F_2_ hybrids, respectively.

All hub genes in the Brown module are differentially correlated in at least one subfertile hybrid group relative to fertile hybrids. Figure 4 illustrates the widespread loss of connectivity within the SFAE relative to the fertile F_2_ network. The loss of interactions for four of the module hub genes, *Prkcd*, *Dbnl*, *Ptdss2*, and *Fkbp4*, are shown in figure 4*A*, and the overall pattern of reduced connectivity in the SFAE network is shown in figure 4*B*. The Brown module is significantly enriched for genes involved in cilium organization, and several of the genes with cell component GOs including cilium and/or sperm flagellum were found to interact with the module hub genes (fig. 4*A*).

**Fig. 5.**
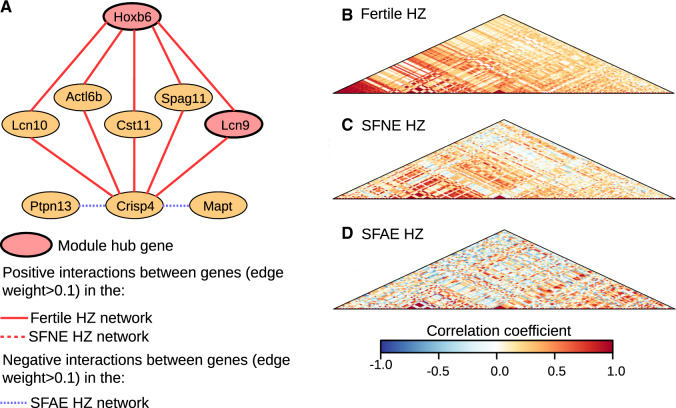
Disrupted interactions of Midnightblue module genes in subfertile HZ hybrids. (*A*) Interactions between Midnightblue module hub genes (red nodes) and genes with functions potentially related to sperm maturation (orange nodes) in HZ hybrids. Gene interactions with an edge-weight exceeding 0.1, as estimated using topological overlap matrices, are indicated using continuous, dashed, and fine-dashed lines for the fertile, SFNE, and SFAE hybrids, respectively. Positive interactions are shown in red and negative interactions are shown in blue. (*B–D*) Coexpression heatmaps showing pairwise correlations between expression values of Midnightblue module genes in fertile, SFNE, and SFAE HZ hybrids, respectively. Heatmaps reveal moderate weakening of gene interactions in the SFNE hybrids and weakening or reversal of interactions in the SFAE HZ hybrids, indicating more severe network disruption in the SFAE hybrid group.

We found some hub genes in significantly preserved modules that nevertheless show disrupted coexpression patterns in subfertile hybrids. For example, the Blue module is significantly preserved in the SFAE F_2_ hybrid group (fig. 3), however, several hub genes show significant loss of interactions in SFAE F_2_ hybrids, including *Hk1*, *Akap1*, *Agpat6*, and *Slain2* (table 3 and supplementary fig. 3*A* and supplementary data 4, Supplementary Material online). The Blue module coexpression heatmaps for fertile and SFAE F_2_ hybrids (supplementary fig. 3*B* and *C*, Supplementary Material online) show weakening of positive correlations in these subfertile hybrids. Meanwhile, the Midnightblue module was preserved in SFNE HZ hybrids, despite showing a lack of preservation in all other subfertile groups (fig. 3). However, two hub genes in this module have changes in interactions in both SFNE and SFAE HZ hybrids (fig. 5*A*). Moreover, an intermediate level of reduced connectivity overall in SFNE HZ hybrids in the Midnightblue module is apparent from visual comparison of the coexpression heatmap of the module compared with fertile hybrids and the more severely disrupted SFAE hybrids (fig. 5*B*–*D*).

## Discussion

Although the DM model provides a well-accepted mechanism for the development of reproductive isolation between diverging lineages, the specific epistatic interactions underlying DMIs remain mostly uncharacterized. A network approach is ideal for identifying complex DMIs, as pairwise interactions between genes are likely to be nonindependent. Here, using hybrids of two house mouse subspecies between which reproductive barriers are incomplete (Britton-Davidian et al. 2005; Albrechtova et al. 2012; Turner et al. 2012), we take a network-based approach to characterize gene interactions in testis of fertile versus subfertile males from two hybrid mapping populations. Combining testis expression data from an F_2_ cross between wild-derived inbred strains (Turner et al. 2014) and offspring of mice wild-caught from a HZ (Turner and Harr 2014) enabled us to generate a consensus fertile network and identify modules of genes that are commonly coexpressed. We identified disruptions in this network in subfertile hybrids at the module and gene level, some of which are associated with specific functions, stages of spermatogenesis, or testis cell types. Integrating our results with previous mapping of subfertility phenotypes and gene expression traits in the same mice (White et al. 2011; Turner and Harr 2014; Turner et al. 2014) reveals candidate pathways and genes for subfertility.

### Network Disruptions in Subfertile Hybrids

Several modules show consistent patterns of poor preservation in subfertile hybrids within both the F_2_ and HZ mapping populations, including modules enriched for cilium organization (Brown), spermatogenesis and the regulation of cell cycle (Blue and Yellow), and DNA repair and chromosome segregation (Green). Of particular note is the Brown module, for which expression is significantly correlated with sperm BCF, a measure of sperm motility which has been associated with fertility in humans (Larsen et al. 2000). The disrupted modules are enriched for genes expressed in spermatogonia (Blue, Green, and Yellow), spermatocytes (Blue, Brown, Green, and Yellow), and spermatids (Blue, Brown, Greenyellow, and Yellow). Hence, our findings suggest that multiple stages of spermatogenesis are impacted by DMIs in both natural and laboratory-bred hybrid mice. These findings are somewhat supported by previous histological analyses of testis defects in the F_2_ hybrid mapping population, which revealed a range of phenotypic defects linked to reduced fertility (Schwahn et al. 2018). Although the majority of these defects could be explained by a failure to complete meiosis I, so potentially implicating genes expressed in primary spermatocytes, possible downstream errors in meiosis II and postmeiotic errors in spermiogenesis were also implicated (Schwahn et al. 2018).

Although most modules enriched for functions potentially related to spermatogenesis show a lack of preservation in at least one of the subfertile hybrid groups, the Magenta module is an exception. This module is relatively well preserved in all subfertile hybrid groups, despite being enriched for similar processes to the poorly preserved Green module (chromosome organization) and despite being enriched with genes expressed in spermatogonia and spermatocytes. This pattern suggests that while several different stages of spermatogenesis are potentially impacted by DMIs; some pathways and processes remain intact.

Patterns of network disruption are broadly similar in the F_2_ and HZ mapping populations, but differences are also evident. The Blue and Yellow modules, for example, showed higher levels of preservation in the F_2_ relative to HZ subfertile hybrids, and several module hub genes showed significant changes in coexpression pattern in only one of the hybrid populations. As noted earlier, power to detect disruptions varies among subfertile classes, due to sample size, but we expect there are also true biological differences, because incompatibility loci are segregating within *musculus* and *domesticus* (Good, Handel, et al. 2008; Larson et al. 2018). Moreover, the HZ mapping population is more genetically and phenotypically diverse (Turner et al. 2012), hence the specific genes driving network disruptions may vary across hybrid populations. It is also possible that severe DMIs might have been purged by selection in the HZ, and are consequently detectable in F_2_ but not HZ hybrids. This phenomenon may explain the lack of evidence for a prominent role for *Prdm9* in the HZ; neither candidate DMI loci based on genomic clines or GWAS for sterility phenotypes overlap *Prdm9* (Janoušek et al. 2012; Turner and Harr 2014). However, ongoing gene flow from pure subspecies populations is likely to counter or subdue the effects of selection, reintroducing even DMI alleles causing severe deleterious effects into the HZ. Cline analysis of HZ populations revealed high variation between markers across the genome in levels of introgression, and linkage disequilibrium between nonadjacent markers in the center of the HZ (Teeter et al. 2007), supporting the influence of selection on gene flow and the presence and prevalence of DMIs.

### Variation in Patterns of Network Disruption

We split hybrid mice with subfertile phenotypes into two broad categories: mice with similar overall patterns of gene expression to those seen in fertile hybrids (SFNE) and those with relatively aberrant patterns of gene expression (SFAE). Unsurprisingly, network disruption appears more severe in the SFAE hybrid groups from both the F_2_ and HZ populations, with fewer modules showing evidence of significant preservation. However, there is evidence for more subtle network disruption in the SFNE hybrids. Two modules are not preserved in the SFNE F_2_ hybrid group (Midnightblue and Greenyellow, fig. 3). In SFNE HZ hybrids, all modules are preserved but intermediate levels of disruption are apparent upon detailed examination of module-specific coexpression patterns within the Midnightblue module (fig. 5).

We identified more fine-scale disruptions in networks by performing differential correlation analysis, which detects genes showing a significant change in coexpression pattern between groups. Genes showing a significant loss or reversal of coexpression patterns were detected in both SFNE and SFAE hybrid groups relative to fertile hybrids, in both the F_2_ and HZ mapping populations, supporting an influence of subtle network disruptions in all subfertile hybrid groups. The 2,800 genes with different interactions in at least one subfertile group include 95 module hub genes, some of which are in relatively well-preserved modules, suggesting hybrid incompatibilities can cause minor or major perturbations in gene interactions. Hence, our findings support varying levels of network disruption within and among subfertile hybrid groups, as expected because sterility loci are segregating within the mapping populations and putative DMIs previously identified show a range of complexity and effect size (White et al. 2011; Turner and Harr 2014; Turner et al. 2014).

### Overlap with Previously Identified Sterility Regions

Several modules, including the Black, Brown, Green, Greenyellow, Red, Purple, and Turquoise modules, have gene content that overlaps significantly with QTT that are associated with a specific *trans*-eQTL hotspot. The majority of these modules show weak (Brown, Green, Greenyellow) or intermediate (Black, Turquoise) preservation in subfertile hybrids, potentially supporting a role for network disruptions involving specific *trans*-eQTL. The Purple and Red modules, however, are well preserved across subfertile hybrid groups and overlap significantly with QTT associated with multiple *trans*-eQTL hotspots. Unlike the more weakly preserved modules, very few of the Purple and Red module genes are expressed during spermatogenesis (Ernst et al. 2019), rather both modules are enriched for genes expressed in Leydig cells. The overall expression of both modules is negatively correlated with both fertility phenotypes, suggesting expression tends to be higher in subfertile relative to fertile hybrids. These observations are consistent with previous reports that genes expressed in somatic cells in testis (i.e., Leydig, Sertoli) have relatively high expression in subfertile hybrids (Turner et al. 2014), likely reflecting reduction/absence of germ cells.

### Candidate DMI Genes

We highlight the following as candidate DMI genes: genes that show a significant loss or reversal in coexpression patterns in subfertile relative to fertile hybrid groups; genes that fall within sterility regions previously identified in the HZ and F_2_ mapping populations; and genes that are highly connected hubs within disrupted modules. The latter are good candidates for large-effect DMIs, as disrupted epistatic interactions involving these genes may have knock-on effects on module-wide gene expression. Moreover, candidate DMIs previously identified in both the F_2_ and HZ mapping populations suggest most DMI loci have more than one incompatible interacting partner locus (Turner and Harr 2014; Turner et al. 2014), as would be expected for hub genes. Module hub genes were found to be significantly more likely than a random sample of testis-expressed genes to fall within GWAS sterility regions, supporting their likely involvement in DMIs. Hub genes previously prioritized as candidate DMI loci include *Nr2c2*, *Zfp711*, *Zfp770*, *Hoxb6*, *Cry2*, and *Gsx1*, all of which lie within *trans*-eQTL hotspots, and *Cyp11a1*, which is located within a GWAS sterility locus on chromosome 9. All of these genes have GO categories that include the regulation of transcription and/or spermatogenesis. The Black module hub gene *Nr2c2* is of particular interest. *Nr2c2* is expressed in mid- and late-stage pachytene spermatocytes and round spermatids (Mu et al. 2004; Ernst et al. 2019) and a lack of expression has been associated with disruptions to late meiotic prophase and consequent delays to spermiogenesis (Mu et al. 2004). The transcription factors *Zfp711* (Green module), *Zfp770* (Green module), *Hoxb6* (Midnightblue module), and *Gsx1* (Turquoise module) are also good candidate loci for large-effect DMIs, as they show a loss of coexpression patterns in subfertile relative to fertile hybrids and are hub genes within modules that show weak (Green and Midnightblue) or intermediate (Turquoise) preservation in subfertile hybrids.

*Trans*-eQTL hotspots and many GWAS sterility loci each contain numerous genes (Turner and Harr 2014; Turner et al. 2014), and honing in on the specific causative genes driving DMIs has been challenging. Genes that lie within candidate regions but lack GOs relating to the regulation of transcription and/or spermatogenesis are likely to have been overlooked. Our study identified several hub genes that lie within previously identified sterility regions yet have not previously been highlighted as likely candidate DMI loci. One such gene is *Prkcd*, a hub gene within the poorly conserved Brown module that lies within a GWAS sterility region on chromosome 14 (Turner and Harr 2014). Although this gene lacks GOs related to spermatogenesis, a knockout study has reported a role for *Prkcd* in male fertility. Specifically, the sperm of male mice lacking *Prkcd* expression have a reduced ability to penetrate the zona pellucida, potentially impairing fertilization (Ma et al. 2015). *Hk1*, a hub gene within the Blue module, lies within a *trans*-eQTL hotspot on chromosome 10 yet also lacks GOs related to spermatogenesis. This gene encodes the enzyme that initiates the glycolysis pathway, which is important for sperm motility (Mori et al. 1998), hence suggesting an important role in male fertility. Other genes that lie within *trans*-eQTL hotspots and/or GWAS sterility regions yet have been overlooked as candidate DMI loci include *Rapgef1* and *Mapkap1*, which are involved in the regulation of signal transduction and establishment of actin cytoskeleton polarity, respectively, *Akap1*, which is known to be involved in meiosis in female mice (Newhall et al. 2006), and *Atr*, which plays a role in preventing DNA damage and is associated with reduced testis weight, abnormal DNA replication and cell cycle in knockout male mice (Murga et al. 2009).

Although several hub genes within the poorly preserved Midnightblue module lie within previously identified sterility regions, only *Hoxb6* has been highlighted as a candidate DMI locus. *Adam28*, *Lcn10*, and *Lcn9* all lie within GWAS sterility regions, and both *Adam28* and *Lcn9* show a loss of positive coexpression patterns in subfertile relative to fertile hybrids. Although all three genes lack GOs relating to spermatogenesis, *Adam28*, *Lcn9*, and *Lcn10* are all known to be expressed in the epididymis, and are likely to be involved in male fertility (Suzuki et al. 2004; Oh et al. 2005). Although our expression data are from testis, we suspect most of these genes are expressed in both tissues as testes are relatively easy to separate from epididymis during dissection. In fertile hybrids, the Midnightblue module hub genes are coexpressed with several genes thought to be involved in sperm maturation, including *Cst11* and *Spag11*, both of which have antimicrobial activity and are thought to be important for sperm maturation in other mammal species (Hamil et al. 2002; Avellar et al. 2007), and *Crisp4*, which is implicated in the acrosome reaction required for the binding of sperm to the zona pellucida (Turunen et al. 2012). Hence disrupted interactions in Midnightblue module hub genes may have an impact on sperm motility and functioning.

Our gene network analysis is largely independent of phenotype data, hence potentially enabling us to identify entirely novel candidate DMI loci. Candidate DMI genes that lie outside of previously identified sterility regions include *Fkbp4* and *Ptdss2*, which are hub genes in the poorly preserved Brown module and show loss of coexpression patterns in subfertile relative to fertile hybrids. Mice lacking *Ptdss2* expression have reduced testis weight and can be infertile (Bergo et al. 2002), whereas the lack of *Fkbp4* expression is associated with abnormal sperm morphology (Hong et al. 2007). *Helq*, a differentially correlated hub gene in the Turquoise module, also lies outside of previously identified sterility regions and is associated with subfertile phenotypes in male mice (Adelman et al. 2013). Finally, *D1Pas1* and *Adam1a*, both hub genes in the Blue module have GOs relating to spermatogenesis and the binding of sperm to the zona pellucida, respectively, yet have not been previously identified as candidates for DMI speciation genes.

### Conclusion

We demonstrate widespread disruptions to gene-interaction networks in association with reduced fertility in hybrid *musculus–domesticus* house mice. Disruptions are variable in magnitude among hybrid mapping populations and appear to affect multiple stages of spermatogenesis, including chromosome segregation and cell cycle, assembly of the sperm flagellum, and sperm maturation. We identify specific candidate DMI genes, several of which fall within previously identified sterility loci and have been previously associated with reduced fertility phenotypes in male mice.

## Materials and Methods

### Microarray and Phenotype Data

We used testis gene expression data from two previous studies, the first included F_2_ hybrid males from a cross between wild-derived inbred strains of *M. m. domesticus* (WSB/EiJ) and *M. m. musculus* (PWD/PhJ) (White et al. 2011; Turner et al. 2014) and the second included first-generation offspring of mice wild-caught in the HZ (Turner and Harr 2014). Gene expression in the testis of hybrid males sacrificed at a similar developmental stage (70±5 days and 9–12 weeks for F_2_ and HZ mice, respectively) was measured using Whole Mouse Genome Microarrays (Agilent), as described in Turner et al. (2014) and Turner and Harr (2014).

To investigate changes in gene expression networks associated with sterility, we first classified individuals as “fertile” versus “subfertile.” As fertility (i.e., ability to father offspring) was not directly measured in F_2_ or HZ individuals, we used two phenotypes to categorize males as fertile or subfertile: relative testis weight (testis weight/body weight) and sperm count (White et al. 2011; Turner et al. 2012). These phenotypes were measured comparably in mice from both mapping populations and have been associated with reduced fertility in multiple studies of *musculus–domesticus* hybrids (Britton-Davidian et al. 2005; reviewed in Good, Dean, et al. 2008; Good et al. 2010); we will henceforth refer to these traits as “fertility phenotypes.” A total of 102 F_2_ and 79 HZ hybrid males have fertility phenotypes that fall within 1 SD of the pure subspecies mean and were categorized as “fertile.” “Subfertile” hybrids, for which one or both of the sterility phenotypes fall outside of the pure subspecies range include 107 F_2_ and 55 HZ males. For a total of 92 F_2_ and 41 HZ hybrids, both sterility phenotypes fall within the pure subspecies range yet at least one of the phenotypes is >1 SD from the pure subspecies mean. These hybrids were categorized as “intermediate phenotype.” Finally, data were available for 32 pure subspecies males: 16 *domesticus* and 16 *musculus*. Eight individuals each of pure *domesticus* and pure *musculus* were offspring of mice wild-caught at the edges of the HZ (Turner and Harr 2014), and the remaining pure subspecies males were from wild-derived inbred strains WSB/EiJ (*domesticus*) and PWD/PhJ (*musculus*), whose expression was reported in Turner et al. (2014). In total, microarray and sterility phenotype data were available for 467 hybrid and 32 pure subspecies males.

### Microarray Data Processing

The Whole Mouse Genome Microarray (Agilent) contains 43,379 probes including 22,210 transcripts from 21,326 genes. We started from raw array data from each study rather than processed expression values, to ensure data sets were comparable in network analyses. Preprocessing of raw expression data was performed in the R package limma (Smyth 2005). Background correction was performed by specifying the “half” setting, which resets intensities that fall <0.5 following background subtraction to 0.5, and by adding an offset of 50. To identify probes with consistently low expression, the 98th percentile of the expression of negative control probes was calculated and only probes that were at least 10% brighter than this background expression level in at least 10% of sampled individuals were retained, reducing the data set to a total of 36,896 probes. The quantile method was used to normalize expression between arrays.

As the expression data set includes data generated within different laboratories and over different time periods, nonbiological systematic bias or “batch effects” must be considered. We adjusted for known batch effects using the empirical Bayes framework implemented via the ComBat function (Johnson et al. 2007). We also tried detecting and adjusting for hidden batch effects using the SVA R package (Leek and Storey 2007) and obtained similar results, with several of the detected surrogate variables clustering the data by the known batches. Adjusting for the batch effect may result in losing potential heterogeneity in gene expression between the F_2_ and HZ mapping populations. Nevertheless, this effect should be equal for both fertile and subfertile phenotypes, and should therefore have minimal impacts on observed patterns of network disruption in subfertile hybrids. Variation in gene expression across individuals was summarized using PCA, as implemented using the prcomp function in R (R Core Team 2018), which uses a singular value decomposition of the centered and scaled data matrix, and extreme outliers were identified visually and removed prior to downstream analyses. Batch-corrected and normalized expression data are available on the Gene Expression Omnibus (GEO) (GSE136886).

### Weighted Gene Coexpression Network Analysis

We used The WGCNA R package (Langfelder and Horvath 2008) to identify groups of genes, “modules,” showing similar patterns of expression within and across the F_2_ and HZ fertile hybrid groups. As network analysis is computationally intensive, we further filtered the data set prior to network construction. Specifically, the connectivity of each probe was estimated using the softConnectivity function, which is available within the WGCNA R package (Langfelder and Horvath 2008) and calculates the sum of the adjacency of each probe to all other probes within the data set. The median connectivity was calculated and probes with above-average connectivity within fertile hybrids were retained, resulting in a final data set of 18,411 probes representing 10,531 genes. We used the blockwiseConsensusModules function to perform signed network construction and identify consensus modules across the fertile F_2_ and HZ data sets, assigning each probe to a single module. Briefly, this process involved calculating a pairwise coexpression matrix for each of the F_2_ and HZ fertile groups, in which coexpression is estimated using Pearson correlation values. Raising the coexpression matrix to a defined soft-threshold power introduces scale-free topology, in which a small proportion of nodes (hub genes) have a large number of connections within the network (Zhang and Horvarth 2005). Such scale-free topology is thought to be a fundamental property of most biological networks (Barabasi and Albert 1999). Network topology analysis was performed for a range of soft-threshold values, and an optimal soft-threshold value of five was chosen as the lowest value at which median connectivity reached a low plateau. The coexpression matrix was raised to this soft-threshold power to create an adjacency matrix, which was then converted to a topological overlap matrix (TOM). The TOM describes the network interconnectivity or coexpression between each pair of genes in relation to all others in the network. A consensus TOM was then used to cluster genes using average linkage hierarchical clustering. A dynamic tree cutting algorithm was used to cut the clustering tree, so defining consensus modules of similarly expressed genes. The deepSplit and minimum module size parameters were set to 0 and 50, respectively, and the ME distance threshold was set to 0.2 to merge similar modules.

Module eigengenes are defined as the first principal component describing the expression of a given module. To determine whether specific modules are more or less expressed in fertile versus subfertile individuals, Pearson correlations were computed between the eigengene of each module and each of the two fertility phenotypic trait values (relative testis weight and sperm count). To determine whether the expression of specific modules was associated with sperm motility, we took phenotype data available for a subset (*n* = 121) of HZ hybrid males from Turner et al. (2012) and used Pearson correlation tests to estimate the significance of relationships between the expression of each module and the following sperm motility traits: track velocity (VCL), smoothed path velocity (VAP), straight line velocity (VSL), amplitude of latitudinal head displacement (ALH), and beat-cross frequency (BCF).

### GO Enrichment Analysis

We tested for functional enrichment within each module on the basis of GO overrepresentation analysis with Benjamini–Hochberg *P*-value adjustment (Boyle et al. 2004), performed using the enrichGO function from the clusterProfiler R package (Yu et al. 2012). We performed this analysis using two “gene universes” as the background: firstly, a gene universe consisting of the 17,514 genes with above-background levels of expression in the testis and secondly, a universe consisting of all 21,200 genes associated with the G4122F microarray (Agilent Whole Mouse Genome Microarray), as established using the Gene Expression Omnibus entry for that platform (Edgar et al. 2002; Barrett et al. 2012). GO terms associated with more than 10 and fewer than 500 genes in the gene universe were available for assignment. As there were few clear differences in the identity and significance of GO annotations using the full data set of genes versus the testis-expressed subset, results are presented for the analysis using the testis-expressed gene universe only.

### Network Preservation between Fertile and Low-Fertility Hybrid Groups

To identify gene interactions which are present in fertile hybrids and disrupted in hybrids with low fertility, we tested for preservation of modules from the fertile network in intermediate and subfertile phenotype hybrids. Levels of genetic variability are likely to vary between the HZ and F_2_ mapping populations, as the F_2_ hybrids were created through crosses of inbred *domesticus* and *musculus* strains, whereas the HZ population was created through crosses of mice caught wild in the HZ. As incompatibility loci are likely to be segregating in natural *domesticus* and *musculus* populations, the presence or absence of specific sterility loci is likely to differ between the mapping populations. We therefore tested for module preservation in subfertile hybrids independently within the F_2_ and HZ populations. As the PCA revealed a strong association between the low-fertility phenotype and variation along PC1 (see Results), we further split the subfertile individuals into those clustering together with the fertile individuals along PC1 and those with a PC1 score that falls outside of the fertile range (−91.25 to 49.33; see fig. 1). We refer to these groupings as SFNE and SFAE. To explore whether the lack of preservation for several modules in the subfertile phenotypes was exclusive to the SFAE group, we tested for module preservation between the fertile and each of the SFNE and SFAE groups within the F_2_ and HZ populations.

We used the statistical frameworks implemented in the WGCNA and NetRep R packages to estimate module preservation (Langfelder and Horvath 2008; Ritchie et al. 2016). Both of these permutation-based approaches use the seven preservation metrics developed by Langfelder et al. (2011). The modulePreservation function (WGCNA package, 500 permutations) was used to generate *Z*_summary_ scores, which combine several preservation statistics that compare the density and pattern of connections within modules and between data sets. *Z*_summary_ scores of ≥10, 2–10, and <2 indicate strong, weak, and a lack of module preservation between data sets, respectively (Langfelder et al. 2011). Modules were ranked according to their relative preservation using the median rank statistic, which is based on the *Z*_summary_ score and module size (Langfelder et al. 2011).

In addition, we used the NetRep R package (Ritchie et al. 2016) to test the significance of all seven of Langfelder’s statistics summarizing the preservation of modules between test and discovery data sets. If one or more of the NetRep statistics was found to be nonsignificant, then this was considered evidence for a lack of significant module preservation in subfertile hybrids. The *Z*_summary_ scores and least significant NetRep statistics for each module are presented in supplementary table 1, Supplementary Material online.

### Differential Correlation Analysis

Genes showing significantly different patterns of pairwise coexpression in the subfertile relative to the fertile hybrids were identified using the R package DGCA (McKenzie et al. 2016). Once again, differential correlation analyses were performed independently for each of the F_2_ and HZ populations, and for each of the SFNE and SFAE hybrid groups. The median log-fold change in pairwise coexpression was estimated for each gene in each of the 15 modules, and the significance of median log-fold change values was estimated using 100 permutations.

### Identification of Module Hub Genes

Two methods were used to identify hub genes. First, genes with a Module Membership (kME) ≥ 0.85 were identified as hub genes, where kME represents the Pearson correlation between the expression of an individual gene and the ME (Horvath and Dong 2008). Second, connectivity statistics including the average number of neighbors, which describes the average connectivity of nodes in a module, and the network density, which summarizes the overall module connectivity, were calculated independently for fertile F_2_ and HZ hybrids, using Cytoscape v3.7.1 (Shannon et al. 2003). The top five most connected genes within each of the F_2_ and HZ networks were also classified as hub genes for each module. We then used permutation tests to determine whether the module hub genes were significantly more likely than a random subset of genes to fall within either GWAS sterility regions or *trans*-eQTL hotspots. We performed 10,000 random draws of 281 genes from the full set of testis-expressed genes, as well as from the subset of genes that were assigned to 1 of the 15 modules in the consensus fertile network. We then calculated the 95% quantiles for the number of genes within GWAS sterility region genes and the number of genes within eQTL hotspots for these randomly drawn gene sets, and assumed significant enrichment of GWAS sterility region or eQTL hotspot genes if the values observed in the module gene data set equaled or exceeded these 95% quantiles. 

## Supplementary Material

msaa002_Supplementary_Data
