# Supplementary material for: Disrupted Gene Networks in Subfertile Hybrid House Mice
Source: Mol Biol Evol. 2020 Jan 12;37(6):1547–62. doi: 10.1093/molbev/msaa002 (PMC7253214; doi:10.1093/molbev/msaa002)
Supplement: msaa002_Supplementary_Data [file msaa002_supplementary_data.zip › msaa002-suppl_data/SupplementaryFigure_3.pdf]

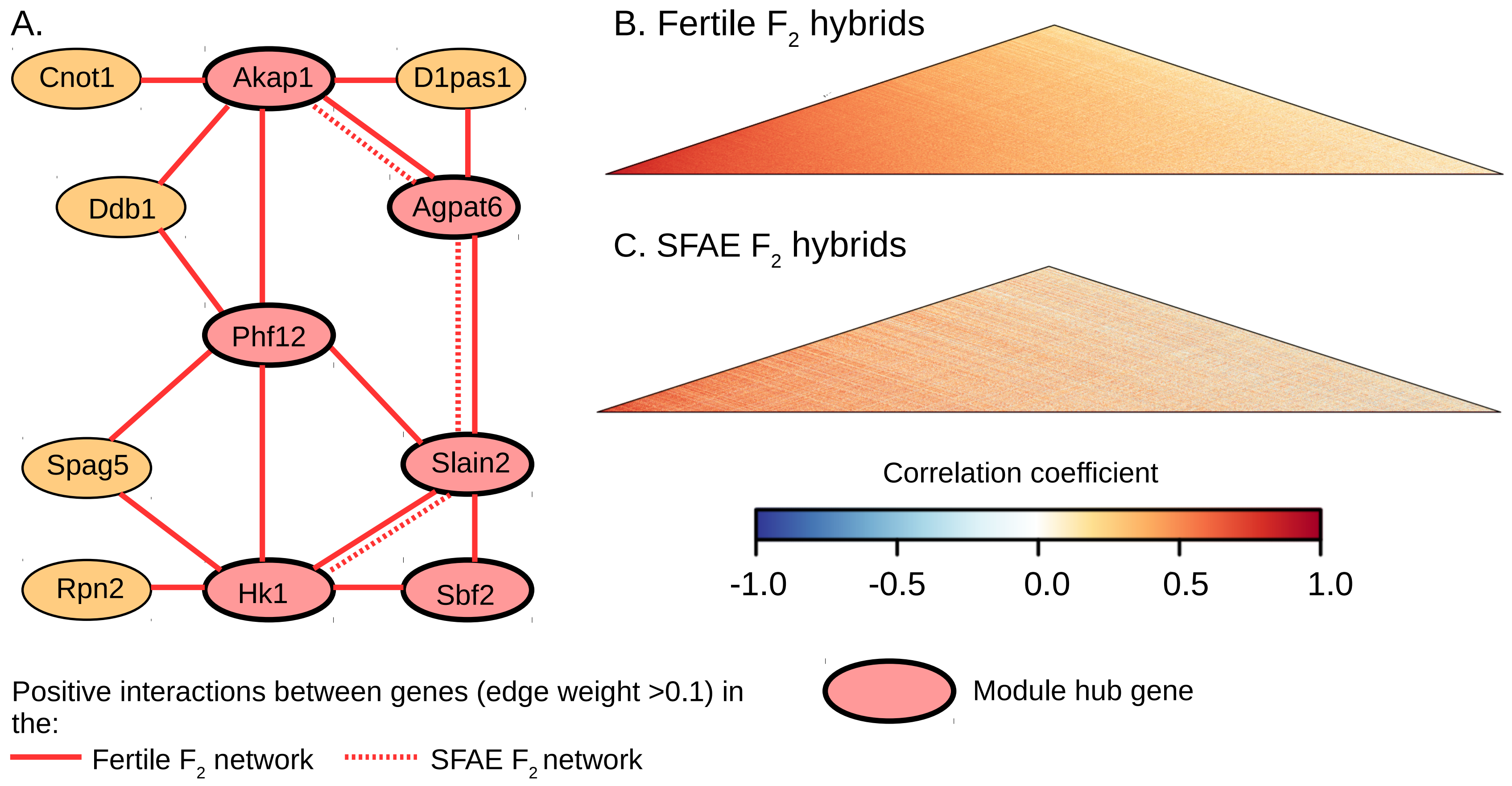
A.
Cnot1
Akap1
D1pas1
Ddb1
Agpat6
Phf12
Spag5
Slain2
Rpn2
Hk1
Sbf2
B. Fertile F2 hybrids
C. SFAE F2 hybrids
Correlation coefficient
-1.0
-0.5
0.0
0.5
1.0
Positive interactions between genes (edge weight >0.1) in the:
Fertile F2 network
SFAE F2 network
Module hub gene
