# Supplementary material for: Disrupted Gene Networks in Subfertile Hybrid House Mice
Source: Mol Biol Evol. 2020 Jan 12;37(6):1547–62. doi: 10.1093/molbev/msaa002 (PMC7253214; doi:10.1093/molbev/msaa002)
Supplement: msaa002_Supplementary_Data [file msaa002_supplementary_data.zip › msaa002-suppl_data/SupplementaryTable1.pdf]

Supplementary Table 1. Module preservation statistics; Statistics indicating a lack of significant module preservation are highlighted in bold.

Module	HZ – all low fertility		F_2 – all low fertility		HZ – SFNE only		F_2 – SFNE only		HZ – SFAE only		F_2 – SFAE only		HZ – Intermediate phenotype		F_2 – Intermediate phenotype	
	Z score*	NetRep[#]	Z score*	NetRep[#]	Z score*	NetRep[#]	Z score*	NetRep[#]	Z score*	NetRep[#]	Z score*	NetRep[#]	Z score*	NetRep[#]	Z score*	NetRep[#]
Black	30.0	**0.99660**	20.0	**1.00000**	64.0	0.00009	34.0	0.00009	20.0	0.00009	22.0	0.00009	41.0	0.00009	59.0	0.00009
Blue	**4.3**	**1.00000**	19.0	0.00150	31.0	0.00009	53.0	0.00009	**4.4**	**0.99970**	25.0	0.00009	28.0	0.00009	51.0	0.00009
Brown	**9.7**	**0.55464**	**7.9**	0.00009	32.0	0.00009	38.0	0.00009	**2.8**	**0.20918**	**7.1**	**0.09789**	32.0	0.00009	35.0	0.00009
Cyan	**9.3**	**0.99380**	**7.9**	**0.90861**	17.0	0.00009	14.0	0.00009	**9.5**	**0.27687**	**9.0**	**0.04900**	12.0	0.00009	14.0	0.00009
Green	**11.0**	**0.13049**	**4.7**	0.00280	17.0	0.00009	27.0	0.00009	**8.8**	**0.85901**	10.0	0.00009	30.0	0.00009	35.0	0.00009
Greenyellow	**9.6**	**1.00000**	**2.8**	**1.00000**	25.0	0.00009	17.0	**1.00000**	**1.7**	**1.00000**	**8.2**	**0.36126**	25.0	0.00009	26.0	0.00009
Magenta	39.0	0.00009	28.0	0.00009	110.0	0.00009	91.0	0.00009	32.0	0.00009	33.0	0.00009	79.0	0.00009	120.0	0.00009
Midnightblue	**5.4**	**0.99570**	**4.8**	**1.00000**	14.0	0.00040	11.0	**0.58614**	**2.0**	**0.95540**	**6.7**	**0.97690**	12.0	0.00009	12.0	0.00009
Pink	11.0	0.00009	**8.9**	0.00130	24.0	0.00009	35.0	0.00009	**8.7**	0.00430	13.0	0.00009	20.0	0.00009	32.0	0.00009
Purple	25.0	**0.86161**	48.0	0.00009	27.0	0.00009	32.0	0.00009	34.0	0.00710	52.0	0.00009	21.0	0.00009	35.0	0.00009
Red	40.0	0.00009	72.0	0.00009	37.0	0.00009	48.0	0.00009	44.0	0.00009	67.0	0.00009	42.0	0.00009	44.0	0.00009
Salmon	**4.2**	**0.54144**	**6.8**	**0.00140**	15.0	0.00009	17.0	0.00009	**7.3**	**0.01560**	**8.7**	0.00009	15.0	0.00009	17.0	0.00009
Tan	11.0	**0.95250**	**9.9**	**0.01720**	25.0	0.00009	27.0	0.00009	13.0	0.00009	**9.7**	**0.03620**	30.0	0.00009	28.0	0.00009
Turquoise	27.0	**1.00000**	24.0	**0.63124**	63.0	0.00009	40.0	0.00009	22.0	0.00009	31.0	0.00009	51.0	0.00009	52.0	0.00009
Yellow	**5.0**	**0.55444**	**8.0**	**0.99640**	27.0	0.00009	29.0	0.00500	**5.2**	**0.67963**	23.0	0.00009	25.0	0.00009	33.0	0.00009

*$Z_{summary}$ scores, which summarize seven preservation statistics comparing the density and pattern of connections within modules and between datasets (Langfelder *et al.* 2011).

#the least significant p-value for Langfelder's seven preservation metrics compared between test and discovery datasets in NetRep (Ritchie *et al.* 2016).
