# Supplementary material for: Disrupted Gene Networks in Subfertile Hybrid House Mice
Source: Mol Biol Evol. 2020 Jan 12;37(6):1547–62. doi: 10.1093/molbev/msaa002 (PMC7253214; doi:10.1093/molbev/msaa002)
Supplement: msaa002_Supplementary_Data [file msaa002_supplementary_data.zip › msaa002-suppl_data/SupplementaryTable2.pdf]

Supplementary Table 1. The percentage of the total genes within each module that show significantly different coexpression patterns in subfertile relative to fertile hybrids. Subfertile hybrids are categorized according to the mapping population and the overall expression pattern.

Module	Percentage genes showing significantly differential correlation				
	F2:SFAE	F2:SFNE	HZ:SFAE	HZ:SFNE	At least one subfertile group
Black	6.26	6.82	0.42	0.00	11.82
Blue	13.86	14.42	4.01	0.64	26.32
Brown	44.51	2.35	1.65	0.24	45.76
Cyan	2.94	7.84	0.00	2.94	10.78
Green	7.16	33.45	1.90	9.62	43.29
Greenyellow	6.15	41.10	5.05	1.76	46.59
Magenta	2.49	1.00	0.66	0.00	3.65
Midnightblue	8.89	22.22	2.22	0.00	28.89
Pink	15.59	3.50	9.46	2.63	25.74
Purple	0.23	1.15	0.23	0.23	1.84
Red	0.00	0.84	0.00	0.42	1.12
Salmon	2.96	4.93	4.43	0.99	11.82
Tan	14.38	7.19	4.38	5.00	25.00
Turquoise	1.88	29.74	0.35	0.58	31.20
Yellow	3.32	22.83	8.90	16.83	41.26
Total Network	11.08	14.83	2.88	3.06	27.53
