# Supplementary figures and images for: Disrupted Gene Networks in Subfertile Hybrid House Mice
Source: Mol Biol Evol. 2020 Jan 12;37(6):1547–62. doi: 10.1093/molbev/msaa002 (PMC7253214; doi:10.1093/molbev/msaa002)

A. F<sub>2</sub> hybrids

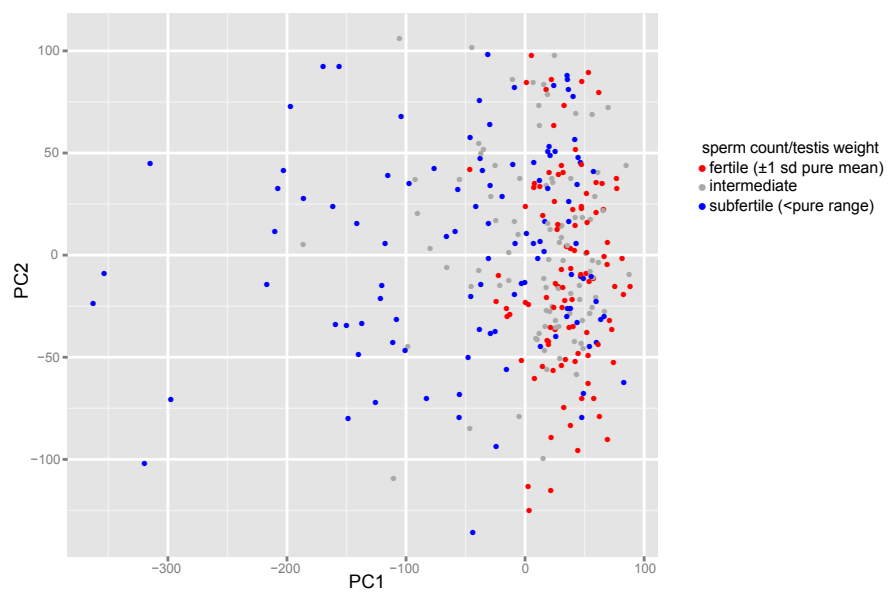

B. Hybrid zone mice

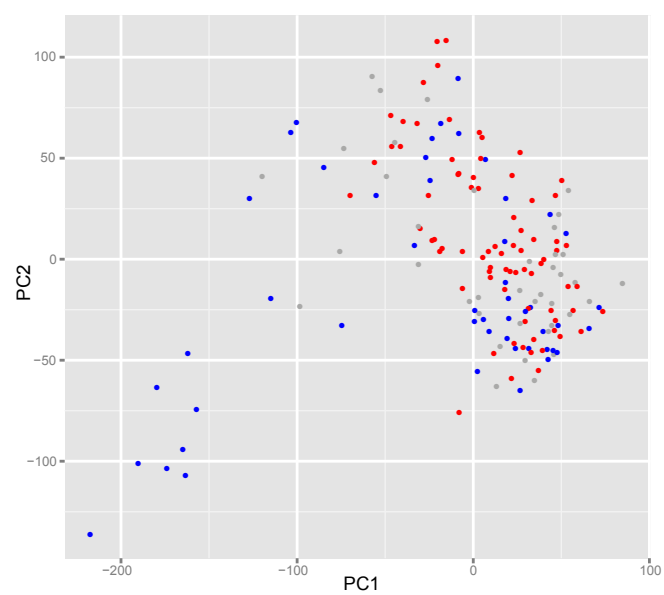

C. PC1 loadings

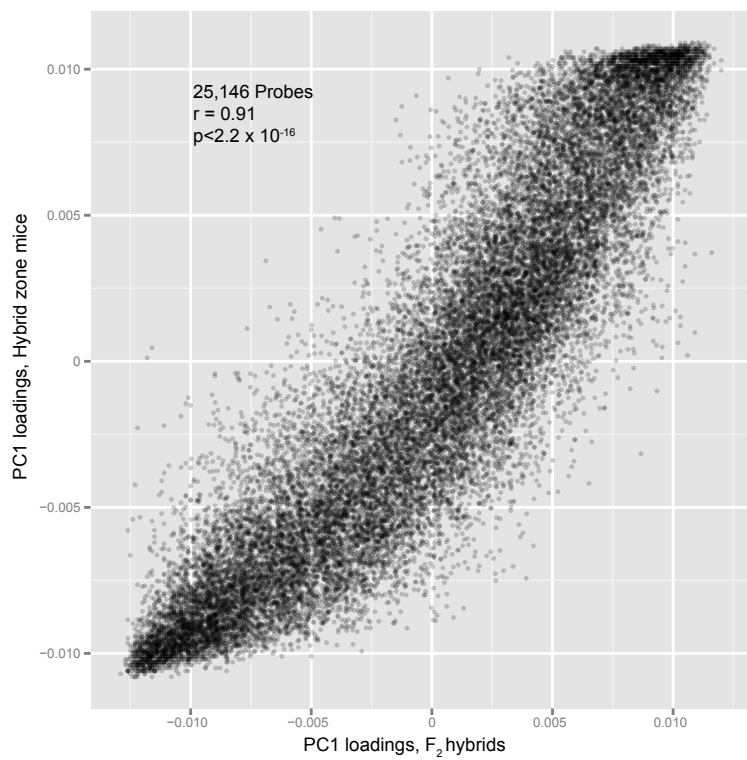

Supplement: msaa002_Supplementary_Data [file msaa002_supplementary_data.zip › msaa002-suppl_data/SupplementaryFigure1.pdf]

A.

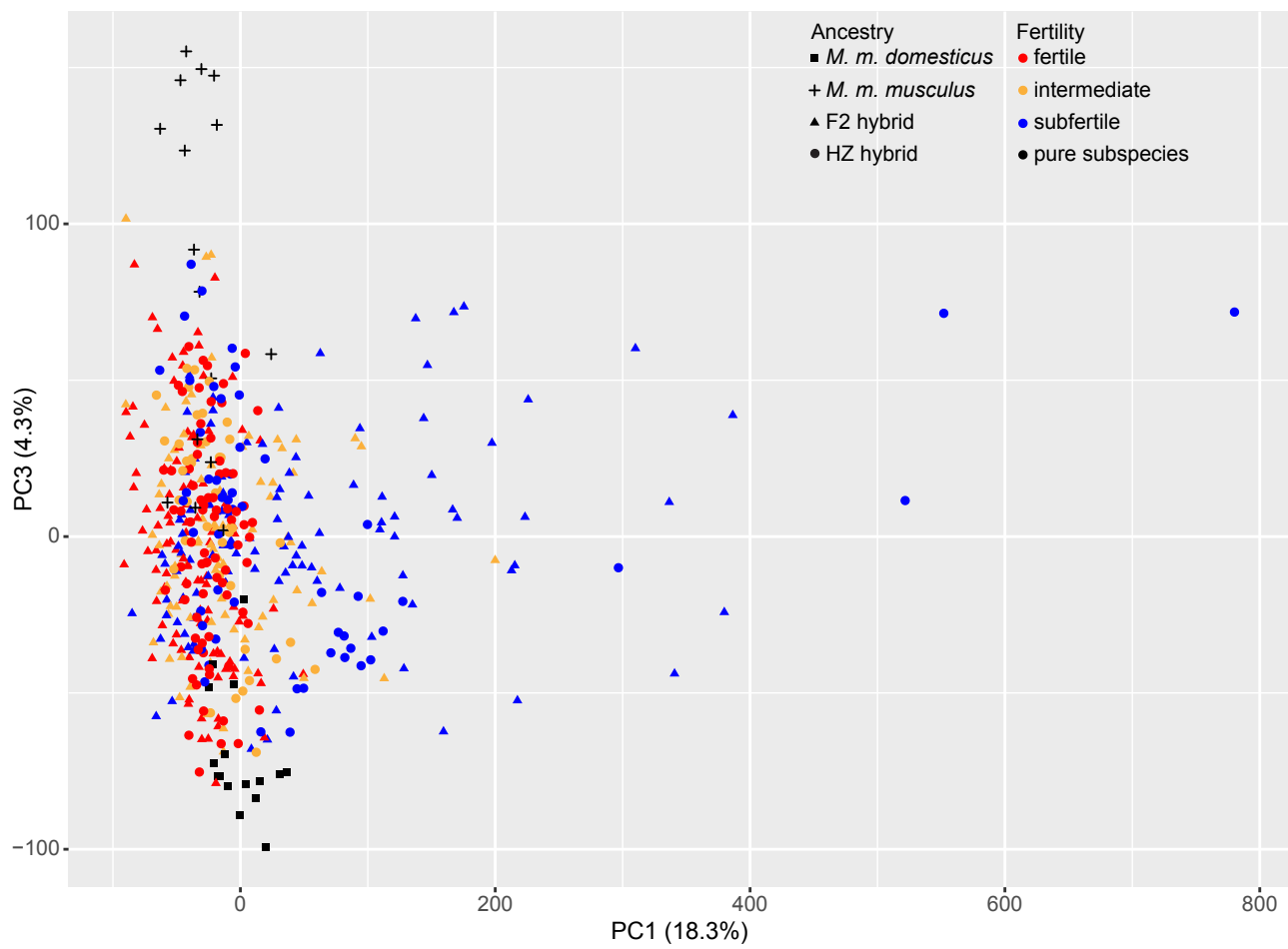

B.

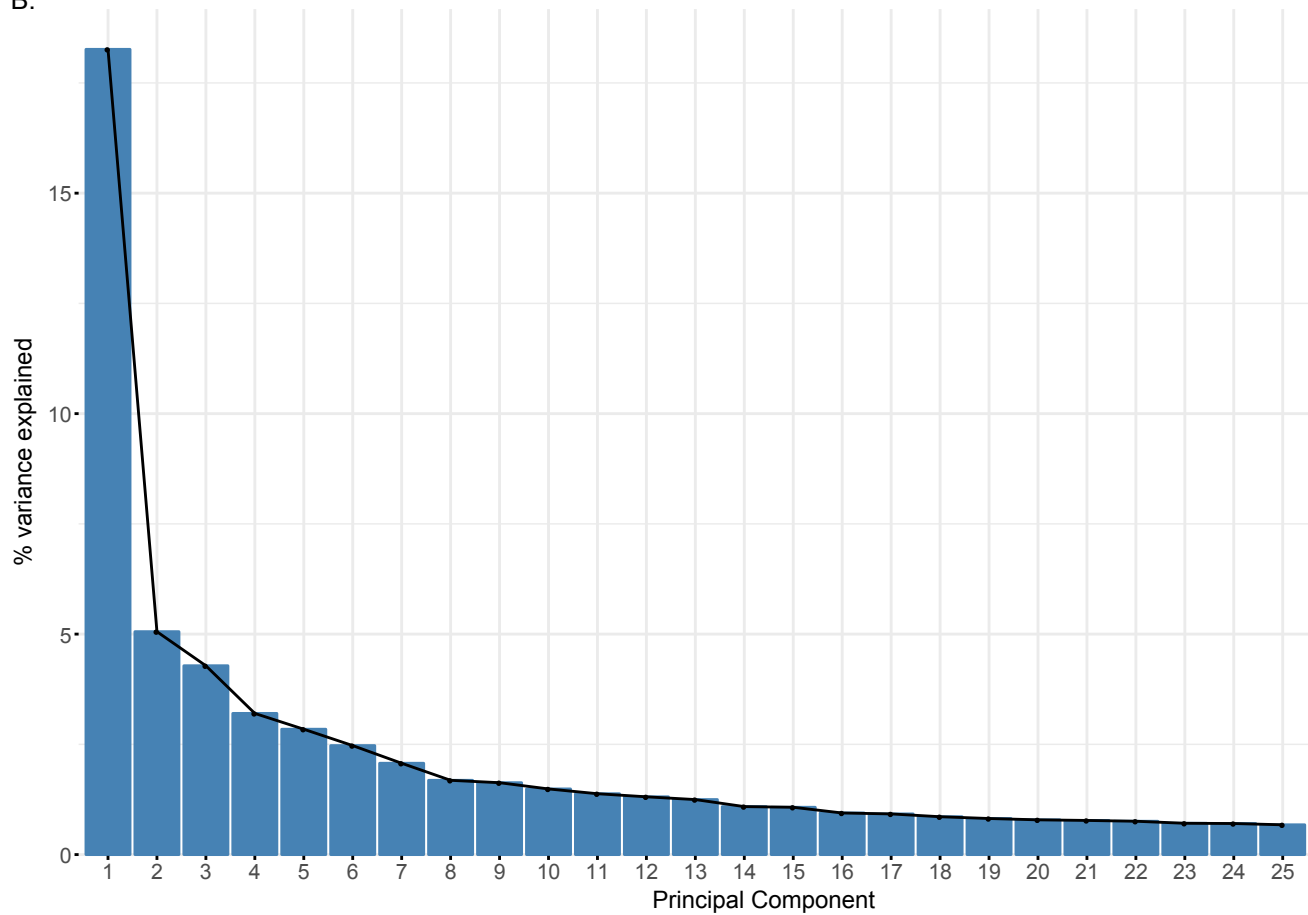

Supplement: msaa002_Supplementary_Data [file msaa002_supplementary_data.zip › msaa002-suppl_data/SupplementaryFigure2.pdf]
